# Supplementary material for: Feasibility of progressive sit-to-stand training among older hospitalized patients
Source: PeerJ. 2015 Dec 17;3:e1500. doi: 10.7717/peerj.1500 (PMC4690357; doi:10.7717/peerj.1500)
Supplement: Supplemental Information 3 [file peerj-03-1500-s003.docx]

Level of STAND in hospital and at home for each set.

|  | In-hospital | | | | | | | | | At home | | | | | | | | | 2 set completed at 8-12 RM | | 1 set completed at 8-12 RM | | Reason for stopping | |
| --- | --- | --- | --- | --- | --- | --- | --- | --- | --- | --- | --- | --- | --- | --- | --- | --- | --- | --- | --- | --- | --- | --- | --- | --- |
|  | Set 1 | | | Set 2 | | | Set 3 | | | Set 1 | | | Set 2 | | | Set 3 | | | Hospital | Home | Hospital | Home | Hosp | Home |
| ID | Level | Reps | Kg | Level | Reps | Kg | Level | Reps | Kg | Level | Reps | Kg | Level | Reps | Kg | Level | Reps | Kg |  |  |  |  |  |  |
| 1 | 6 | 10 | 6 | 7 | 10 | 0 | 8 | 11 | 6 | 8 | 10 | 6 | 8 | 10 | 6 | 8 | 10 | 6 | **√** | **√** | **√** | **√** |  |  |
| 2 | 3 | 8 |  | 3 | 8 |  | . | . |  | . | . |  | . | . |  | . | . |  | **√** | . | **√** | . | MF | DWV |
| 3 | 5 | 10 |  | 5 | 10 |  | . | . |  | 6 | 12 | 4 | 6 | 12 | 10 | 6 | 12 | 10 | **√** | **√** | **√** | **√** | MF |  |
| 4 | 3 | 10 |  | 4 | 12 |  | 5 | 12 |  | 5 | 12 |  | 6 | 12 | 4 | 6 | 12 | 8 | **√** | **√** | **√** | **√** |  |  |
| 5 | 5 | 7 |  | 4 | 7 |  | . | . |  | 5 | 8 |  | 5 | 8 |  | . | . |  | **÷** | **√** | **÷** | **√** | MF | MF |
| 6 | . | . |  | . | . |  | . | . |  | . | . |  | . | . |  | . | . |  | . | . | . | . | BR | BR |
| 7 | 3 | 6 |  | 3 | 3 |  | . | . |  | 3 | 10 |  | 3 | 10 |  | 3 | 10 |  | **÷** | **√** | **÷** | **√** | BP |  |
| 8 | 5 | 10 |  | 6 | 10 | 6 | 6 | 10 | 10 | 6 | 10 | 18 | 6 | 10 | 26 | 7 | 10 |  | **√** | **√** | **√** | **√** |  |  |
| 9 | 5 | 5 |  | 3 | 8 |  | . | . |  | 5 | 10 |  | 5 | 10 |  | 5 | 10 |  | **÷** | **√** | **√** | **√** | KP |  |
| 10 | 5 | 10 |  | 6 | 10 | 2 | . | . |  | 6 | 10 | 4 | 6 | 10 | 4 | . | . |  | **√** | **√** | **√** | **√** | MF | MF |
| 11 | 3 | 6 |  | . | . |  | . | . |  | 5 | 8 |  | 5 | 10 |  |  |  |  | **÷** | **√** | **÷** | **√** | DP |  |
| 12 | 3 | 5 |  | 1 | 10 | 4 | 1 | 10 | 6 | 3 | 10 |  | 4 | 10 |  | 4 | 10 |  | **√** | **√** | **√** | **√** |  |  |
| 13 | 1 | 10 | 3 | . | . |  | . | . |  | 3 | 10 |  | 3 | 10 |  | . | . |  | **÷** | **√** | **√** | **√** | DP | MF |
| 14 | 5 | 10 |  | 5 | 10 |  | . | . |  | 5 | 12 |  | 6 | 10 | 4 | . | . |  | **√** | **√** | **√** | **√** | MF | MF |
| 15 | 6 | 10 | 6 | 7 | 7 |  | 7 | 7 |  | 7 | 7 |  | 7 | 10 |  | . | . |  | **÷** | **÷** | **√** | **√** |  | MF |
| 16 | 5 | 10 |  | 5 | 15 |  | . | . |  | 5 | 15 |  | . | . |  | . | . |  | **√** | **÷** | **√** | **√** | KPA | KPA |
| 17 | 4 | 8 |  | 4 | 8 |  | . | . |  | . | . |  | . | . |  | . | . |  | **√** | . | **√** | . | MF | DWV |
| 18 | 5 | 10 |  | . | . |  | . | . |  | 5 | 10 |  | . | . |  | . | . |  | **÷** | **÷** | **√** | **√** | MF | MF |
| 19 | 6 | 10 | 6 | . | . |  | . | . |  | 6 | 12 | 8 | 7 | 3 |  | . | . |  | **÷** | **÷** | **√** | **√** | HA | MF |
| 20 | 5 | 6 |  | 5 | 8 |  | . | . |  | 5 | 10 |  | 5 | 11 |  | . | . |  | **÷** | **√** | **√** | **√** | MF | MF |
| 21 | 7 | 10 |  | 7 | 10 |  | . | . |  | 7 | 10 |  | 7 | 10 |  | 8 | 8 | 4 | **√** | **√** | **√** | **√** | MF |  |
| 22 | 5 | 10 |  | 6 | 10 | 4 | 6 | 10 | 8 | . | . |  | . | . |  | . | . |  | **√** | . | **√** | . |  | DD |
| 23 | 5 | 10 |  | 6 | 10 | 6 | 6 | 12 | 10 | . | . |  | . | . |  | . | . |  | **√** | . | **√** | . |  | DWV |
| 24 | 4 | 12 |  | 4 | 6 |  | . | . |  | 3 | 12 |  | 3 | 12 |  | 3 | 12 |  | **÷** | **√** | **√** | **√** | MF |  |

**√**=yes; **÷**=no

MF: Muscular fatigue; DWV: doesn’t want to visit; BR: doesn’t want to leave bed; BP: back pain before testing; KP: knee pain before testing; DP: dyspnea; KPA: knee pain during activity – wating to get alloplastics; HA: headache; DD: deterioration disease
